# Supplementary material for: Targeted Next-Generation Sequencing in a Large Cohort of Genetically Undiagnosed Patients with Neuromuscular Disorders in Spain
Source: Genes (Basel). 2020 May 11;11(5):539. doi: 10.3390/genes11050539 (PMC7288461; doi:10.3390/genes11050539)
Supplement: Supplementary file 1 [file genes-11-00539-s001.pdf]

Supplementary data. Relevant clinical, pathological and genetic data in each patient.

ID PATIENT	Serum CK	Sex/ Age at Onset	Key pathological features on muscle biopsy	Clinical data/ Clinical orientation	Candidate variants
P1	<200 U/L	M / Prenatal	Central cores	Polyhydramnios, severe neonatal hypotonia. Limb girdle weakness and facial hypomimia / CM	NM_000540.2(RYR1): [c.3362A>G p. Tyr1121Cys]; [c.6891G>C p. Lys2297Asn]
P2	NA	M / Congenital	NA	NA	No candidate variants identified
P3	NA	F / Adulthood	NA	Limb girdle muscle weakness / LGMD	NM_006790.2(MYOT): c.179C>G p. Ser60Cys
P4	1844 U/L	M / 16 yr.	NA	Quadriceps hypertrophy / NA	NM_001177634.2(DAG1): [c.337_340del p. Gln113Alafs*20; c.2228A>C p. Lys743Thr]
P5	<200 U/L	M / 2 yr.	NA	Weakness and myasthenic crises / CMS	NM_001244710.1(GFPT1): c.1501G>A p. Val501Ile
P6	NA	M / 2 yr.	NA	Distal contractures and skin biopsy compatible with Bethlem myopathy / COL6-related	NM_001848.2(COL6A1): c.362A>G p. Lys121Arg

				myopathy	
P7	Normal	M / 8 months	Type 1 fiber predominance	Plagiocephaly, microcephaly and hypotonia / NA	NM_001376.4(DYNC1H1): c.10378G>C p. Ala3460Pro
P8	Normal	M / NA	NA	NA	NM_000070.2(CAPN3): c.2257G>A p. Asp753Asn
P9	<200 U/L	F / 5 yr.	NA	Ptosis / CMS	No candidate variants identified
P10	NA	F/ NA	NA	NA	NM_001267550.1 (TTN): c.101212C>T p. Arg33738Cys
P11	NA	F/ NA	NA	NA	NM_000426.3(LAMA2): c.5445+1G>A p.?
P12	<200 U/L	M / 1 yr.	NA	Ptosis and proximal weakness / CMS	No candidate variants identified
P13	NA	M / NA	NA	NA / LGMD	NM_001267550.1(TTN): [c.48751G>A p. Asp16251Asn]; [c.38829del p. Val12944Cysfs*3]
P14	NA	F/ NA	NA	NA	No candidate variants identified
P15	NA	M / Congenital	Dystrophic pattern	Axial and peripheral hypotonia /	NM_000426.3(LAMA2): c.3976C>T p. Arg1326*

			Partial merosin deficiency	*LAMA2*-related CMD	
P16	374 U/L	M / 54 yr.	Myofibrillar myopathy	Leg and joint pain / Myofibrillar Myopathy	NM_001927.3(DES): c.326A>G p. Lys109Arg
P17	NA	M / NA	NA	NA	No candidate variants identified
P18	958 U/L	F / 43 yr.	Type 1 fiber predominance Atrophic fibers	Paresis of scapular waist with left predominance / NA	NM_000540.2(RYR1): c.6856C>G p. Leu2286Val
P19	NA	F/ NA	NA	NA	NM_012470.3(TNPO3): c.2771del p.*924Cysfs*16
P20	559 U/L	F / 68 yr.	Fiber size variability, central nuclei and inflammatory infiltrates	Posterior distal weakness, cardiomyopathy / NA	NM_001267550.1(TTN): c.9763C>A p. Pro3255Thr
P21	NA	F / 1 month	NA	Axial hypotonia, patellar reflexes present but diminished, abnormal synaptic transmission / CMS	No candidate variants identified
P22	3661 U/L	F / 24 yr.	Dystrophic pattern. Absence of *DYSF* expression on immunohistochemistry	Limb-girdle muscle weakness / Dysferlinopathy	NM_003494.3(DYSF): [c.5594del p. Gly1865Alafs*101]; [c.5594del p. Gly1865Alafs*101]

P23	602 U/L	F / 45 yr.	NA	Gait abnormalities since age 45 / NA	No candidate variants identified
P24	295 U/L	M / 24 yr.	Myopathic changes, rimmed vacuoles	Axial muscle atrophy, severe weakness in lower limbs / NA	NM_000070.2(CAPN3): c.1910del p. Pro637Hisfs*25
P25	x6-10-fold	F / 2nd decade of life	Dystrophic pattern	NA / LGMD	NM_003494.3(DYSF): [c.855del p. Val286Trpfs*2]; [c.855del p. Val286Trpfs*2]
P26	2217 U/L	M / 18 yr.	Dystrophic pattern	NA / LGMD	NM_012470.3(TNPO3): c.2771del p.*924Cysfs*16
P27	NA	F / Congenital	Central cores	NA / CM	NM_000540.2(RYR1): c.14761T>C p. Phe4921Leu
P28	380 U/L	M / 12 months	Dystrophic pattern	NA	No candidate variants identified
P29	NA	M / 49 yr.	Internal nuclei in 80% of fibers	Proximal muscle weakness in upper and lower limbs / Centronuclear Myopathy	NM_005677.3(COLQ): c.1289A>C p. Tyr430Ser
P30	136 U/L	M / 11 yr.	NA	NA	No candidate variants identified
P31	> 1300 U/L	M / 53 yr.	Small protein aggregates	Distal muscle weakness / NA	NM_058246.3(DNAJB6): c.962C>T p. Ser321Leu

P32	159 U/L	M / 74 yr.	Unspecific myopathic changes	Asymmetric distal weakness in lower limbs with myalgias. Anterior tibial weakness / NA	NM_001267550.1(TTN): [c.107889del p. Lys35963Asnfs*9]; [c.79316G>A p. Arg26439His]
P33	112 U/L	F / 9 yr.	NA	Limb-girdle muscle weakness / NA	NM_001244710.1(GFPT1): [c.44C>T p. Thr15Met]; [c.44C>T p. Thr15Met]
P34	122 U/L	M/ 20 months	NA	Hypotonia, axial muscle weakness / CM	NM_000540.2(RYR1): c.6503T>A p. Val2168Glu
P35	<200 U/L	M / Neonatal	Central nuclei	Hypotonia / CM	NM_000252.2(MTM1): c. (?_445) _ (*1548_?)
P36	270 U/L	F / Neonatal	Dystrophic changes, COL VI Reduction in muscle and skin biopsy	Hypotonia, congenital torticollis, limb- girdle weakness and multiple retractions / COL6-related myopathy	NM_001849.3(COL6A2): c.901-2A>G p.?
P37	NA	F / 14 yr.	Vacuoles	Limb-girdle muscle weakness / NA	NM_001244710.1(GFPT1): c.686-2A>G p.?
P38	NA	M / NA	NA	NA / CM	NM_000540.2(RYR1): c.14524G>A p. Val4842Met
P39	749 U/L	M / 4 yr.	NA	Reduced collagen VI expression in skin biopsy / COL6-related	NM_001848.2(COL6A1): c.1056+1G>A p.?

				myopathy	
P40	537 U/L	M/ 37 yr.	NA	Asymmetric involvement of the lumbar paravertebral muscles, pelvic girdle and lower limbs / NA	No candidate variants identified
P41	189 U/L	F / 79 yr.	NA	Distal anterior weakness / NA	No candidate variants identified
P42	1862 U/L	F / 14 yr.	Dystrophic pattern. Macrophage infiltration	Limb-girdle muscle weakness / LGMD	NM_001039885.2(FKRP): [c.163G>C p. Glu55Gln]; [c.826C>A p. Leu276Ile]
P43	NA	M / 30 yr.	Dystrophic pattern, rimmed vacuoles and protein aggregates	Asymmetric extensor finger weakness in upper limbs at onset, followed by selective deltoid involvement, with relative sparing of leg muscles / NA	NM_001267550.1(TTN): [c.54710T>C p. Leu18237Pro]; [c.95372G>A p. Gly31791Asp]
P44	NA	F / NA	NA	Limb-girdle muscle weakness, limb contractures/ NA	NM_001848.2(COL6A1): c.1056+1G>A p.?
P45	NA	M / NA	NA	Distal muscle weakness, pes cavus / NA	NM_001605.2(AARS): c.2192C>T p. Ser731Leu
P46	<200 U/L	M / First year of	NA	Fatigability, weakness / CMS	No candidate variants identified

		life			
P47	103 U/L	M / Neonatal	Myopathic changes with central nuclei	NA / Centronuclear Myopathy	NM_000540.2(RYR1): [c.9157C>T p. Arg3053*]; [c.13672C>T p. Arg4558Trp]
P48	Normal	F / Congenital	Multiminicores	NA / CM	NM_000540.2(RYR1): [c.11798A>G p. Tyr3933Cys]; [c.10097G>A p. Arg3366His]
P49	NA	M / NA	NA	NA	No candidate variants identified
P50	NA	M / Congenital	NA	Hypotonia, diplegic facies, palpebral ptosis, limb-girdle weakness / CMS	NM_000080.3(CHRNE): c.865C>T p. Leu289Phe
P51	NA	M / NA	NA	Asymptomatic hyperCKemia / NA	No candidate variants identified
P52	NA	F / 33 yr.	NA	Symmetric and bilateral lower limb hypertrophy / NA	No candidate variants identified
P53	NA	F / Congenital	Congenital Fiber Type Disproportion	NA	NM_213674.1(TPM2): c.415_417del p. Glu139del
P54	NA	F / NA	NA	NA	No candidate variants identified

P55	x2-fold	M / 20 yr.	Isolated fibers with rimmed vacuoles	NA	NM_007126.4(VCP): c.1202A>G p. Asn401Ser
P56	NA	M / 13 yr.	NA	NA / CMS	No candidate variants identified
P57	NA	M / 2 yr.	Normal	Limb-girdle weakness, hyperlordosis, asymmetric muscle hypoplasia / NA	No candidate variants identified
P58	1100 U/L	F / 30 yr.	Myopathic changes	Limb-girdle muscle weakness / LGMD	No candidate variants identified
P59	NA	M / NA	NA	NA	No candidate variants identified
P60	1604 U/L	M / Infancy	Myopathic changes	NA	No candidate variants identified
P61	NA	F / NA	NA	Fatigability / CMS	No candidate variants identified
P62	NA	M / NA	NA	NA	No candidate variants identified
P63	<200 U/L	M / Neonatal	Myopathic changes	Hypotonia, muscular weakness and joint hyperlaxity / COL6-related myopathy	No candidate variants identified

P64	1148 U/L	M / 44 yr.	Rimmed vacuoles	Hereditary inclusion body myositis / NA	NM_001267550.1(TTN): c.53096G>A p. Arg17699His
P65	2286 U/L	M / 18 months	Dystrophic pattern, Partial merosin deficiency	NA / *LAMA2*-related CMD	NM_000426.3(LAMA2): [c.2584T>C p. Cys862Arg]; [c.2584T>C p. Cys862Arg]
P66	91 U/L	M / 50 yr.	Internal nuclei	Cardiomyopathy and axial weakness / NA	NM_001267550.1(TTN): c.44280_44281+14del p. Lys14760Asnfs*48
P67	NA	M / NA	NA	NA	No candidate variants identified
P68	x20-fold	M / 27 yr.	Dystrophic pattern	Distal and asymmetric limb-girdle muscle dystrophy / NA	NM_001267550.1(TTN): c.86483A>G p. Gln28828Arg
P69	456 U/L	F / 41 yr.	Myopathic changes	Distal muscle atrophy, fat degeneration of tibialis anterior / NA	NM_001128227.2(GNE): [c.1853T>C p. Ile618Thr]; [c.1853T>C p. Ile618Thr]
P70	1597 U/L	M / 37 yr.	Muscle fiber regeneration, abundant ring fibers and nuclear clumps	NA	No candidate variants identified
P71	x3-fold	M / 4th decade of life	Myopathic changes	Asymmetric calf atrophy / NA	NM_000540.2(RYR1): c.6406C>A p. Arg2136Ser

P72	Normal	M/ First decade of life	Internal Nuclei	NA / CM	NM_001005361.2(DNM2): c.1105C>T p. Arg369Trp
P73	NA	F / NA	Dystrophic pattern	NA	No candidate variants identified
P74	Normal	F / 51 yr.	Normal	NA / CMS	No candidate variants identified
P75	NA	M / Congenital	Predominance of type 1 fibers	Clubfoot with walking difficulties / NA	No candidate variants identified
P76	Normal	F / Congenital	Myopathic changes and predominance of type 1 fibers	NA / CM	NM_001267550.1(TTN): [c.37284del p. Val12429Cysfs*518]; [c.88601C>G p. Pro29534Arg]
P77	27 U/L	F / 6 yr.	NA	NA / CMS	No candidate variants identified
P78	Normal	M / 3rd decade of life	NA	Cardiac conduction abnormalities, myopathic facies / NA	No candidate variants identified
P79	173 U/L	M / NA	NA	NA	NM_002047.3(GARS): c.1421G>C p. Arg474Pro
P80	781 U/L	F / 10 yr.	NA	HyperCKemia / NA	NM_004369.3(COL6A3): [c.7447A>G p. Lys2483Glu]; [c.7447A>G p. Lys2483Glu]

P81	NA	M / 13 yr.	NA	Myopathy and epilepsy / NA	No candidate variants identified
P82	NA	M / Congenital	NA	Dry skin with hyperkeratosis, joint hyperlaxity. Bilateral and symmetric atrophy. Atrophy with fatty infiltration of the muscle groups of both shoulder girdles / COL6-related myopathy	NM_001848.2(COL6A1): c.717+4A>G p. [=, Ile239fs*30]
P83	NA	F / NA	Predominance of type 1 fibers, ring fibers	NA / Distal myopathy	No candidate variants identified
P84	NA	F / NA	NA	NA	NM_152393.3(KLHL40): [c.1174T>C p. Trp392Arg]; [c.515T>C p. Leu172Pro]
P85	NA	M / NA	NA	NA	No candidate variants identified
P86	Normal	M / Congenital	NA	Congenital hypotonia and hyperlaxity / NA	No candidate variants identified
P87	<200 U/L	M / Neonatal	Myopathic changes	Hypotonia, joint hyperlaxity / NA	No candidate variants identified
P88	NA	M / NA	NA	NA	NM_020451.2(SELENON): [c.1189C>T p. Gln397*];

					[c.404-1G>A p.?]
P89	Normal	M / Congenital	Myopathic changes	Hypomimia, severe scoliosis, respiratory insufficiency, axial hypotonia / NA	NM_001100.3(ACTA1): c.772C>G p. Arg258Gly
P90	NA	M / NA	NA	NA / Centronuclear Myopathy	No candidate variants identified
P91	NA	M / NA	NA	NA	No candidate variants identified
P92	<200 U/L	M / First year of life	Normal	Motor delay / CMS	No candidate variants identified
P93	Normal	F / NA	Congenital fiber type disproportion	Scoliosis, myopathic facies, generalized hypotonia / NA	NM_152263.2(TPM3): c.733A>G p. Arg245Gly
P94	NA	M / Congenital	Central cores	NA / CM	NM_000540.2(RYR1): c.14818G>A p. Ala4940Thr
P95	502 U/L	M / 57 yr.	Myopathic changes	Distal atrophy of the lower limbs / Distal myopathy	NM_001267550.1(TTN): c.101345C>T p. Thr33782Ile
P96	Normal	M / Infancy	Myopathic changes and type 1 fiber predominance	Slowly progressive facioscapulohumeral-peroneal syndrome / NA	No candidate variants identified

P97	<200 U/L	M / Neonatal	Congenital fiber type disproportion	Hypotonia / CM	NM_001100.3(ACTA1): c.925C>T p. Pro309Ser
P98	NA	M / NA	Reduction of dystrophin (immunostaining after DMD variant identification)	NA	NM_004006.2(DMD): c.473A>C p. Asn158Thr
P99	Normal	M / 38 yr.	Internal nuclei	NA / CM	NM_001005361.2(DNM2): c.1106G>A p. Arg369Gln
P100	Normal	M / 70 yr.	Rimmed vacuoles and protein aggregates	Muscle weakness / NA	NM_006790.2(MYOT): c.164C>T p. Ser55Phe
P101	NA	M / 50 yr.	NA	Marked trunk weakness and atrophy of paravertebral musculature / NA	No candidate variants identified
P102	538 U/L	M / 69 yr.	NA	Distal myopathy of finger extensors / NA	No candidate variants identified
P103	Normal	M / First decade of life	Neurogenic pattern	NA	NM_001265592.1(PLEKHG5): [c.2989_2990del p. Gly997Leufs*16]; [c.2294del p. Leu765Argfs*79]
P104	x2-fold	M / 3th decade of life	Type 1 fiber predominance	NA / COL6-realted myopathy	NM_004369.3(COL6A3): [c.7447A>G p. Lys2483Glu];

					[c.7447A>G p. Lys2483Glu]
P105	994 U/L	M / 38 yr.	Myopathic changes, rimmed vacuoles	Selective atrophy of right radial carpal extensors and left tibialis anterior / NA	NM_001267550.1(TTN): c.100018C>A p. Gln33340Lys
P106	NA	M / NA	Predominance of type 1 fibers, ring fibers	NA / Myofibrillar myopathy	NM_001267550.1(TTN): c.56557C>T p. His18853Tyr
P107	NA	M / NA	NA	NA / Nemaline myopathy	NM_001271208.1(NEB): [c.10583G>A p. Arg3528His]; [c.25143A>G p.= (p. Gln8381Gln)]
P108	87 U/L	M / Infancy	Central cores	Exercise intolerance / NA	NM_000540.2(RYR1): c.14761T>C p. Phe4921Leu
P109	Normal	M / 60 yr.	Internal nuclei	NA / Centronuclear Myopathy	NM_000540.2(RYR1): c.9148G>A p. Val3050Ile
P110	NA	M / NA	NA	NA	NM_001927.3(DES): c.407T>A p. Leu136His
P111	x3-fold	M / 4th decade of life	Normal	Limb muscle weakness, hyperCKemia / NA	NM_001267550.1(TTN): c.51679G>A p. Ala17227Thr
P112	NA	M / NA	NA	Fatigability / CMS	NM_005055.4(RAPSN): [c.264C>A p. Asn88Lys]; [c.603C>A p. Ser201Arg]

P113	Normal	M / 4th decade of life	Normal	NA	No candidate variants identified
P114	529 U/L	M / 32 yr.	Myopathic changes	Hypertrophic myopathy with muscle hypertrophy and cramps / NA	No candidate variants identified
P115	500 U/L	M / 63 yr.	Normal	Calf hypertrophy, unspecific muscle involvement / NA	No candidate variants identified
P116	Normal	F / Congenital	Normal	Generalized muscle weakness, reported consanguinity / CMS	NM_001039523.2(CHRNA1): [c.257G>A p. Arg86His]; [c.257G>A p. Arg86His]
P117	NA	M / 31 yr.	Cores and cytoplasmic bodies	HyperCKemia, ptosis, arched palate / NA	NM_000540.2(RYR1): c.487C>T p. Arg163Cys
P118	<200 U/L	F / Neonatal	Congenital fiber type disproportion	Hypotonia / CM or CMS	NM_173660.4(DOK7): [c.1124_1127dup p. Ala378Serfs*30]; [c.54+25_55-38del p.?]
P119	NA	F / 35 yr.	Protein aggregates	NA	No candidate variants identified
P120	x3-fold	M / 5th decade of life	Myopathic changes	NA	NM_001267550.1(TTN): c.43986T>G p. Asp14662Glu

P121	100 U/L	M / Neonatal	Myopathic changes	Hypotonia, progressive severe scoliosis / NA	No candidate variants identified
P122	<200 U/L	F / 1 yr.	Cores and protein aggregates	Motor delay / NA	No candidate variants identified
P123	<200 U/L	F / 18 months	Fiber size variability	Fatigability / CMS	NM_005677.3(COLQ): [c.640G>T p. Glu214*]; [c.1289A>C p. Tyr430Ser]
P124	120 U/L	M / Neonatal	Dystrophic changes, COL VI reduction in muscle and skin biopsy	Hypotonia, congenital torticollis, limb- girdle weakness and multiple retractions / COL6-related myopathy	No candidate variants identified
P125	78 U/L	M / 1 yr.	Myopathic changes	Hypotonia, muscle weakness / Centronuclear Myopathy	NM_001005361.2(DNM2): c.1102G>A p. Glu368Lys
P126	NA	F / NA	NA	NA	NM_000080.3(CHRNE): c.488C>T p. Ser163Leu
P127	NA	F / NA	NA	NA / CMS	No candidate variants identified
P128	531 U/L	F / 60 yr.	Unspecific changes	Proximal lower limb weakness	No candidate variants identified
P129	707 U/L	F / 79 yr.	Normal	Facial weakness, limb-girdle	No candidate variants identified

				muscle weakness / NA	
P130	NA	F / 25 yr.	Dystrophic pattern	Limb girdle muscle weakness / LGMD.	NM_000070.2(CAPN3): [c.1706T>C p. Phe569Ser]; [c.1099G>A p. Gly367Ser]
P131	145 U/L	F / 50 yr.	Normal	Significant pelvic and scapular weakness / LGMD related to *TTN*	NM_001267550.1(TTN): [c.80941C>T p. Arg26981Trp]; [c.33838C>T p. Pro11280Ser]
P132	NA	F / 55 yr.	NA	Fatigability, proximal weakness of lower limbs / LGMD	No candidate variants identified
P133	NA	F / NA	NA	NA	NM_006790.2(MYOT): c.179C>G p. Ser60Cys
P134	NA	F / 56 yr.	NA	Myopathy with marked involvement of the paravertebral and lower limb muscles / NA	No candidate variants identified
P135	350 U/L	M / Neonatal	NA	Hypotonia, COL VI reduction in skin biopsy / COL6-related myopathy	NM_001848.2(COL6A1): [c.817A>T p. Lys273*]; [c.817A>T p. Lys273*]
P136	76 U/L	F / Neonatal	Central cores	Hypotonia / CM	NM_000540.2(RYR1): c.14693T>C p. Ile4898Thr
P137	875 U/L	M / NA	Myopathic changes	Proximal weakness of pelvic girdle	NM_001927.3(DES): c.49_54dup p.

				/ LGMD	Thr17_Phe18dup
P138	317 U/L	F / 58 yr.	Dystrophic pattern	Marked weakness of paravertebral musculature resulting in significant camptocormia / NA	NM_001267550.1(TTN): [c.63625C>T p. Arg21209*]; [c.49801G>T p. Val16601Leu]
P139	850 U/L	F / 42 yr.	Normal	HyperCkemia / NA	No candidate variants identified
P140	NA	F / 32 yr.	NA	Ptosis, diplopia, fatigability, muscle weakness and nasal voice / CMS	NM_005055.4(RAPSN): c.493G>A p. Val165Met
P141	28 U/L	F / Infancy	Internal nuclei	Limb girdle muscle weakness / LGMD	No candidate variants identified
P142	1121 U/L	F / 19 yr.	Myopathic changes	NA / First misdiagnosed as myasthenia gravis	NM_213599.2(ANO5): [c.191dup p. Asn64Lysfs*15]; [c.2141C>G p. Thr714Ser]
P143	Normal	F / NA	Type1 fiber predominance	Facial weakness, distal and paraspinal weakness / NA	NM_182914.2(SYNE2): c.11671-1G>C p.?
P144	429 U/L	M / Congenital	Dystrophic pattern	Muscle weakness, hypotonia / NA	NM_001849.3(COL6A2): c.874G>A p. Gly292Ser

P145	<200 U/L	M / Neonatal	Myopathic changes	NA / CM	No candidate variants identified
P146	92 U/L	M / 15 yr.	NA	Asymmetric limb girdle weakness / NA	NM_000070.2(CAPN3): c.2243G>A p. Arg748Gln
P147	Normal	M / 16 yr.	NA	Hyperlordosis, generalized muscle weakness / NA	No candidate variants identified
P148	<200 U/L	M / Prenatal	Myopathic changes	Arthrogryposis Multiplex Congenital / NA	NM_005199.4(CHRNG): [c.459dup p. Val154Serfs*24]; [c.459dup p. Val154Serfs*24]
P149	3800 U/L	M / 7 yr.	NA	HyperCKemia with eosinophilia from age 7 / NA	No candidate variants identified
P150	3718 U/L	M / Neonatal	Dystrophic pattern, partial merosin deficiency	Muscle weakness / *LAMA2*-related CMD	No candidate variants identified
P151	Normal	F / Fifth decade of life	NA	Fatiguability, dysphonia, slight ptosis / CMS	No candidate variants identified
P152	NA	M / NA	NA	Generalized muscle weakness, scoliosis, facial and neck muscle weakness / NA	NM_001100.3(ACTA1): [c.317T>C p. Leu106Pro]; [c.616+2T>C p.?]
P153	<200 U/L	F / Neonatal	Dystrophic pattern, presence of	Hypotonia and weak suction / CM	NM_000540.2(RYR1):

			cores		c.12187A>G p. Met4063Val
P154	120 U/L	F / Neonatal	Minicores and rods	Muscle weakness / CM	No candidate variants identified
P155	NA	F / NA	NA	NA	NM_170707.2(LMNA): c.117T>G p. Asn39Lys
P156	3500U/L	M / Neonatal	Dystrophic pattern	Muscle weakness / CMD	NM_000426.3(LAMA2): [c.7057C>T p. Arg2353Cys]; [c.7057C>T p. Arg2353Cys]
P157	2300 U/L	F / Neonatal	Dystrophic pattern, partial merosin deficiency	Hypotonia / *LAMA2*-related CMD	No candidate variants identified
P158	<200 U/L	F / Neonatal	Myopathic changes	Facial hypomimia, limb girdle weakness (>>glutei) and fatigability. No response to mestinon / CMS	NM_000334.4(SCN4A): [c.3798G>C p. Glu1266Asp]; [c.3798G>C p. Glu1266Asp]
P159	<200 U/L	F / Neonatal	Central cores	Muscle weakness / CM	NM_000540.2(RYR1): c.14581C>T p. Arg4861Cys
P160	NA	M / NA	NA	NA / CMS	NM_005055.4(RAPSN): [c.264C>A p. Asn88Lys]; [c.264C>A p. Asn88Lys]
P161	146 U/L	M / 50 yr.	Rimmed vacuoles	Limb girdle muscle weakness / NA	NM_007126.4(VCP): c.784A>G p. Thr262Ala

P162	NA	M / NA	NA	Familiar hyperCKemia / NA	No candidate variants identified
P163	NA	M / NA	NA	Pes Cavus / NA	NM_002047.3(GARS): c.262C>G p. Gln88Glu
P164	NA	F / NA	NA	NA	No candidate variants identified
P165	800-1000 U/L	F / Neonatal	Dystrophic pattern	Muscle weakness / CMD or CMS	NM_013334.3(GMPPB): [c.553C>T p. Arg185Cys]; [c.553C>T p. Arg185Cys]
P166	NA	F / 50 yr.	Dystrophic pattern	Respiratory failure, nocturnal hypoventilation / Myopathy	NM_001267550.1(TTN): c.95134T>C p. Cys31712Arg
P167	NA	F / 3 yr.	Myopathic changes	NA / LGMD	NM_000751.2(CHRND): [c.188T>C p. Leu63Pro]; [c.340G>C p. Val114Leu]
P168	NA	F / NA	NA	NA / LGMD	NM_001039885.2(FKRP): [c.283del p. Arg95Alafs*34]; [c.427C>A p. Arg143Ser]
P169	50 U /L	M / neonatal	NA	Hypotonia and joint hyperlaxity / NA	NM_001267550.1(TTN): [c.35678C>G p. Thr11893Ser]; [c.19558_19561del p. Glu6520Tyrfs*25]
P170	Normal	F / Congenital	No remarkable findings	Congenital hypotonia / CM	NM_001267550.1(TTN):

					c.38737G>T p. Glu12913*
P171	NA	F / NA	NA	NA / Symptomatic dystrophinopathy carrier as a first clinical suspicion	NM_000070.2(CAPN3): [c.550del p. Thr184Argfs*36]; [c.1746-20C>G p. ?]
P172	92 U/L	F / NA	Normal	Exercise intolerance / NA	No candidate variants identified
P173	NA	M / 20 yr.	Dystrophic pattern	Cardiomyopathy and limb girdle muscle weakness / NA	NM_001267550.1(TTN): [c.107889del p. Lys35963Asnfs*9]; [c.4646-1G>A p.?]
P174	<200 U/L	M / Prenatal	Type 1 fiber predominance	Arthrogryposis / CM	NM_001039523.2(CHRNA1): [c.119G>A p. Arg40Gln]; [c.257G>A p. Arg86His]
P175	2400 U/L	M / 18 months	Dystrophic pattern, partial merosin deficiency	NA / *LAMA2*-related CMD	NM_000426.3(LAMA2); [c.2584T>C p. Cys862Arg]; [c.3928G>T p. Glu1310*]
P176	2700 U/L	F / 12 yr.	Dystrophic pattern	Limb girdle muscle weakness, Epidermolysis bullosa simplex / Plectinopathy	NM_201380.2(PLEC): [c.5527C>G p. Gln1843Glu]; [c.2594_2596del p. Phe865del]
P177	NA	F / NA	NA	NA / LGMD	NM_213599.2(ANO5): [c.191dup p. Asn64Lysfs*15]; [c.2521C>G p. His841Asp]

P178	<200 U/L	F / Neonatal	Central cores	Hypotonia / CM	NM_000540.2(RYR1): c.14582G>A p. Arg4861His
P179	NA	F / NA	NA	NA	No candidate variants identified
P180	Normal	M / Congenital	Dystrophic pattern	NA	NM_000540.2(RYR1): c.14693T>C p. Ile4898Thr
P181	NA	F / NA	NA	NA	NM_170707.2(LMNA): c.1562G>C p. Gly521Ala
P182	244 U/L	M / Congenital	Moderate fiber size variability and presence of some nuclear clumps	Motor delay and hypotonia / NA	No candidate variants identified
P183	NA	M / NA	NA	NA	NM_000540.2(RYR1): c.8134C>G p. Pro2712Ala
P184	NA	F / NA	Dystrophic pattern	Proximal muscle weakness / NA	NM_001927.3(DES): c.934G>A p. Asp312Asn
P185	NA	M / NA	Dystrophic pattern	Proximal muscle weakness in upper and lower limbs / NA	NM_001267550.1(TTN): [c.43355G>T p. Arg14452Ile]; [c.11362G>A p. Glu3788Lys]
P186	NA	M / NA	NA	NA	NM_001136504.1(SYT2): c.1082A>G p. Asp361Gly
P187	216 U/L	M / 3 yr.	Cores	Myalgia in lower limbs, difficulties	No candidate variants

				in running and climbing stairs / NA	identified
P188	NA	M / NA	NA	Familiar HyperCKemia and ptosis / NA	NM_001267550.1(TTN): [c.43303C>G p. Pro14435Ala]; [c.69821G>A p. Gly23274Asp]
P189	9909 U/L	F / 25 yr.	Dystrophic pattern	NA / LGMD	NM_000540.2(RYR1): [c.14126C>T p. Thr4709Met]; [c.14126C>T p. Thr4709Met]
P190	4222 U/L	M / Neonatal	Dystrophic pattern, partial merosin deficiency	Hypotonia and muscle weakness / *LAMA2*-related CMD	No candidate variants identified
P191	Normal	M / 18 months	NA	Congenital axial hypotonia, / CM	NM_000540.2(RYR1): c.7844G>A p. Arg2615His
P192	NA	F / NA	NA	NA	NM_001267550.1(TTN): [c.91669C>T p. Arg30557*]; [c.55655G>A p. Arg18552His]
P193	NA	M / NA	NA	Distal muscle weakness / NA	NM_006790.2(MYOT): c.179C>T p. Ser60Phe
P194	1400 U/L	F / 28 yr.	NA	NA / LGMD	NM_001267550.1(TTN): c.107517T>G p. Ser35839Arg
P195	NA	F / NA	NA	NA	No candidate variants identified

P196	Normal	F / NA	Myopathic changes	Hypertrophic Cardiomyopathy / NA	NM_000257.2(MYH7): c.2156G>A p. Arg719Gln
P197	NA	F / NA	NA	NA / LGMD	NM_213599.2(ANO5): [c.191dup p. Asn64Lysfs*15]; [c.692G>T p. Gly231Val]
P198	NA	F / NA	NA	Muscle weakness / NA	NM_000080.3(CHRNE): c.1058_1077del p. Leu353Profs*37
P199	1200 U/L	M / 61 yr.	Myopathic changes	Bilateral and posterior symmetric involvement of the thighs / NA	NM_213599.2(ANO5): [c.692G>T p. Gly231Val]; [c.1119+1G>T p.?]
P200	39 U/L	F / Neonatal	Cores	Hypotonia / CM	NM_000540.2(RYR1): c.7111G>A p. Glu2371Lys
P201	Normal	F / Congenital	Normal	Generalized axial hypotonia / CM	No candidate variants identified
P202	<200 U/L	M / Neonatal	NA	Hypotonia / CM	NM_000540.2(RYR1): c.10229C>T p. Pro3410Leu
P203	NA	M / NA	NA	NA / COL6-realted myopathy	NM_001848.2(COL6A1): c.1056+1G>A p.?
P204	NA	M / Prenatal	Fiber size variability	Congenital arthrogryposis / NA	No candidate variants identified

P205	NA	F / Congenital	Mild dystrophic pattern	Hypotonia, arthrogryposis, rigid spine and respiratory distress / NA	NM_000540.2(RYR1): c.6701G>A p. Arg2234His
P206	NA	F / NA	NA	NA / Distal myopathy	NM_000257.2(MYH7): c.4519+1G>C p.?
P207	<200 U/L	F / Neonatal	Nemaline bodies	Facial hypomimia, limb girdle weakness. No response to mestinon / Nemaline myopathy	NM_001271208.1(NEB): [c.21076C>T p. Arg7026*]; [c.1493A>G p. Asp498Gly]
